# Electrical behavior of multi-walled carbon nanotube network embedded in amorphous silicon nitride

**DOI:** 10.1186/1556-276X-6-88

**Published:** 2011-01-17

**Authors:** Ionel Stavarache, Ana-Maria Lepadatu, Valentin Serban Teodorescu, Magdalena Lidia Ciurea, Vladimir Iancu, Mircea Dragoman, George Konstantinidis, Raluca Buiculescu

**Affiliations:** 1 National Institute of Materials Physics, Magurele 077125, Romania; 2 "Politehnica" University of Bucharest, Bucharest 060042, Romania; 3 National Institute for Research and Development in Microtechnologies, Bucharest 023573, Romania; 4 Institute of Electronic Structures and Laser, Foundation for Research and Technology-Hellas, Heraklion 70013, Crete, Greece; 5 University of Crete, Voutes Campus, Heraklion 71003, Crete, Greece

## Abstract

The electrical behavior of multi-walled carbon nanotube network embedded in amorphous silicon nitride is studied by measuring the voltage and temperature dependences of the current. The microstructure of the network is investigated by cross-sectional transmission electron microscopy. The multi-walled carbon nanotube network has an uniform spatial extension in the silicon nitride matrix. The current-voltage and resistance-temperature characteristics are both linear, proving the metallic behavior of the network. The *I*-*V *curves present oscillations that are further analyzed by computing the conductance-voltage characteristics. The conductance presents minima and maxima that appear at the same voltage for both bias polarities, at both 20 and 298 K, and that are not periodic. These oscillations are interpreted as due to percolation processes. The voltage percolation thresholds are identified with the conductance minima.

## Background

The carbon nanotubes (CNTs), either single-walled (SWCNTs) or multi-walled (MWCNTs), have a quasi-1D behavior that results from their nanometric diameters and micrometric lengths [1-6]. While the SWCNT structures correspond to the rolling up of one graphene sheet, the MWCNTs consist of several concentric sheets.

The electrical behavior of SWCNTs is determined by their chirality, either metallic or semiconductor [7]. The longitudinal conductance of a metallic one is quantified, namely, *G *= *nG*_0_, with *G*_0 _= 2*e*^2^/*h *= 77.47 μS and *n *a natural number. The behavior of MWCNTs is metallic if, at least, one sheet has a metallic chirality. A theoretical analysis on the conductance of infinitely long, defect-free MWCNTs shows that the tunneling current between states on different walls is vanishingly small [8], which leads to the quantization of the conductance. In the frame of this model, the authors showed that in a finite nanotube, the interwall conductance is negligible compared to the intrawall ballistic conductance. Abrikosov et al. [9] calculated the electron spectrum of a metallic MWCNT with an arbitrary number of concentric sheets. They calculated the entropy and density of states for an MWCNT and analyzed the tunneling between the nanotube and a metal electrode. The authors proved that measuring the tunneling conductivity at low temperatures, the one-electron density of states can be directly determined. They also give the necessary restrictions on temperature.

Kuroda and Leburton [10] modeled the linear behavior of the *R*-*T *characteristics measured at low field in SWCNTs, by taking into account the mean free paths determined by the interactions of electrons with acoustic and optical phonons. Their results are in good agreement with the data from Refs [11,12]. This model is generalized for MWCNTs in Ref. [13].

Li et al. [14] measured in individual vertical MWCNTs with large diameters very large currents at low bias voltage and they determined a very high conductance, *G *= 490*G*_0_, much higher than the value of 2*G*_0_, predicted in the literature for perfect metallic SWCNTs. They explained this behavior by a multichannel quasiballistic transport of electrons in the inner walls. In Ref. [15], Collins et al., studying the limits of high energy transport in MWCNTs, showed that the nanotubes fail via a series of sharp and equal current steps, in contrast to metal wires that fail continuously and in accelerating mode.

The percolation phenomena in films with MWCNTs are extensively investigated in the literature, related to film composition and thickness, temperature, nanotubes concentration and shape, and so on. The electrical conductivity of oxidized MWCNT-epoxy composites was investigated in Ref. [16]. The MWCNTs were oxidized under both mild and strong conditions. Strong oxidation conditions produce partially damaged nanotubes. Consequently, their conductivity decreases and the percolation threshold increases. On the contrary, the MWCNTs oxidized under mild conditions present a high conductivity, independent of oxidation conditions. The study of the conductivity as a function of film thickness and nanotube volume fraction [17] shows that reducing the film thickness to a value comparable with the MWCNT length, the percolation threshold significantly diminishes. The authors explain this considering that different conductive paths appear with different probabilities in a film of MWCNT embedded in polyethylene.

The MWCNT-PMMA [poly(methyl methacrylate)] composites also exhibit percolation phenomena. The *dc *conductivity increases with increasing the MWCNTs concentration or mass [18-21], a typical percolation behavior. A percolation threshold of 0.4 wt% was reported in Ref. [20]. Using other polymers as a matrix, e.g., polydimethylsiloxane and styrene acrylic emulsion-based polymer, percolation thresholds of 1.5 wt% [22] and 0.23 wt% were found for MWCNTs [23]. The electrical behavior of the composite formed by an MWCNT network embedded in PMMA is explained by a combination of Sheng's fluctuation induced tunneling and 1D variable range hopping models [20]. Percolation in a 2D MWCNT network [24] is strongly influenced by the MWCNT sizes and shape.

In the present letter we report on the electrical behavior of an MWCNT network embedded in amorphous silicon nitride matrix. The sample preparation and microstructure investigations are presented. The voltage and temperature dependences of the current were measured and the current-voltage, conductance-voltage, and resistance-temperature characteristics are discussed. The observed conductance minima are interpreted as voltage percolation thresholds, analogous to those previously observed on nanostructures formed by nanocrystalline silicon dots embedded in amorphous silicon dioxide matrix, and also in nanocrystalline porous silicon [25].

## Experimental

**Figure 1:**
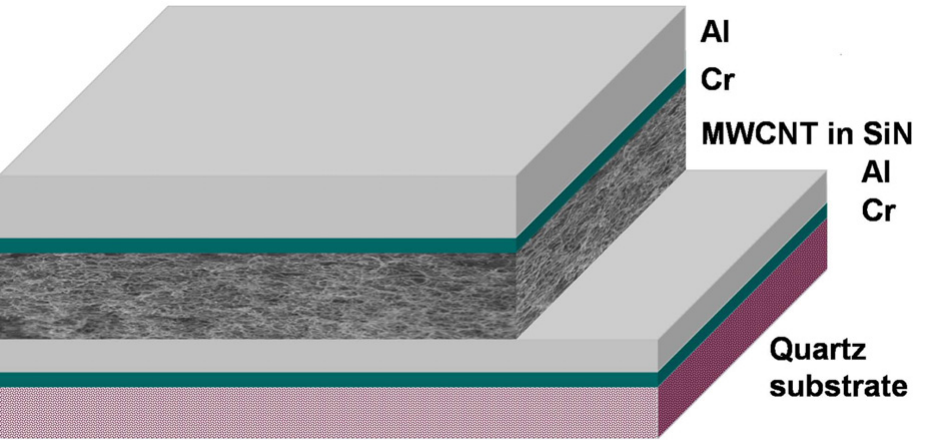
**Sample structure**.

The samples were prepared in a sandwich configuration on a quartz substrate, as presented in Figure 1. The bottom electrode is a 10 nm thin Cr layer as adhesion promoter, and a 1 μm thick Al layer, deposited by "blanket" electron gun evaporation. On this electrode, a solution of MWCNTs (from Nanothinx S. A., Rio Patras, Greece), with tetrahydrofuran (THF = (CH_2_)_4_O) with the ratio MWCNT:THF = 0.22 mg/ml, was deposited by pipetting. After, tetrahydrofuran evaporated, silicon nitride was grown by PECVD to embed the MWCNTs. A 3 minute reactive ion etching in CF_4_/O_2 _mixture was performed to etch the silicon nitride layer, until exposing the top of the nanotubes layer. The final thickness of silicon nitride with MWCNTs is about 8 μm. Then, a 30 minute reactive ion etching in CF_4_/O_2 _mixture was further performed to remove totally the silicon nitride and the nanotubes at one end of the sample, for exposing the bottom electrode. Finally, the top electrode of 10 nm Cr and 2 μm Al layers was deposited by electron gun evaporation, to contact the protruding ends of the nanotubes from the etched silicon nitride.

Cross-sectional transmission electron microscopy (XTEM) investigations were made on a Jeol TEM 200CX instrument. The XTEM specimen was prepared by a conventional method using mechanical polishing and ion thinning in a Gatan PIPS device. Electrical measurements were performed in a Janis CCS-450 cryostat at room temperature (298 K) and low temperature (20 K), using a Keithley 6517A electrometer.

## Results and discussions

**Figure 2:**
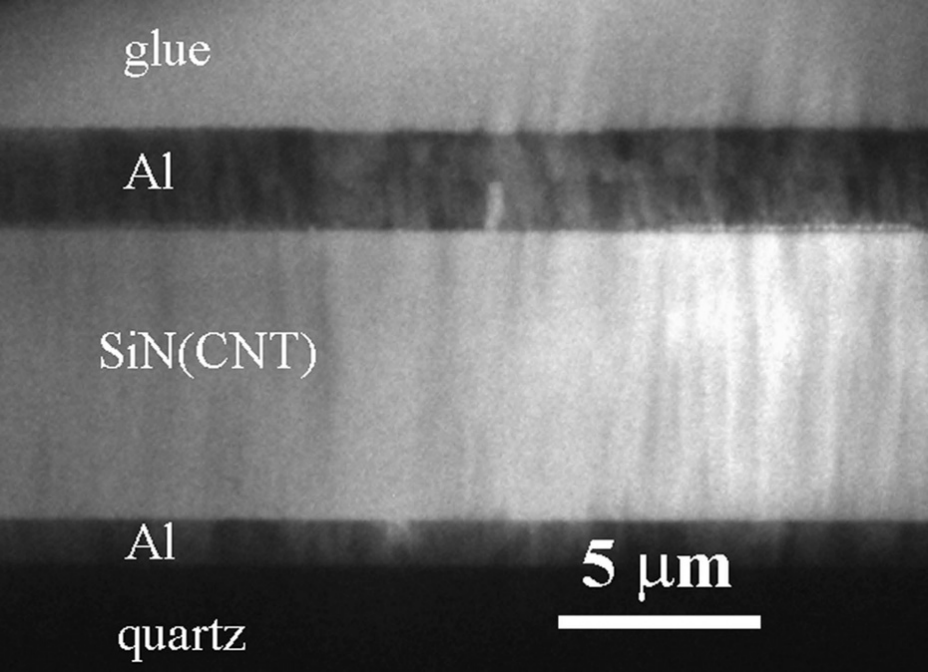
**Low magnification image of a thick area of the XTEM specimen**.

A low magnification image of the cross-section specimen of the Cr/Al/MWCNT-SiN/Cr/Al sandwich sample is presented in Figure 2. It confirms the structure expected from preparation, sketched in Figure 1. One can observe that the MWCNT-SiN layer is about 8 μm in thickness and has an amorphous and homogeneous structure.

**Figure 3:**
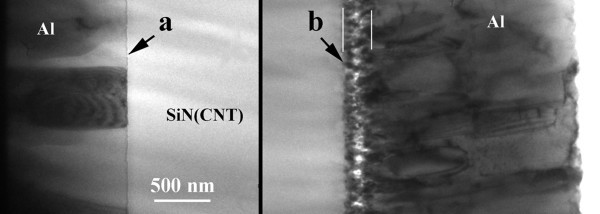
**XTEM images of the electrode/MWCNT-SiN interfaces**. **(a) **bottom interface and **(b) **top interface.

Figure 3 shows the microstructure of interfaces between the electrodes and the MWCNT-SiN layer. The bottom interface (Figure 3a) is neat. The Al crystallites in the electrodes have a columnar morphology. The Cr layer deposited on quartz is too thin to be seen in this image. The top electrode interface looks different compared with the bottom one (Figure 3b). At this interface, the aluminum layer starts with small nanometric crystallites, which are extended about 200 nm in the thickness of the electrode. Then the structure becomes columnar with big crystallites similar to those in the bottom electrode. This difference is most probably induced by the irregularities created by etching the top surface of the MWCNT-SiN layer, and the presence of the few nm thin Cr layer.

**Figure 4:**
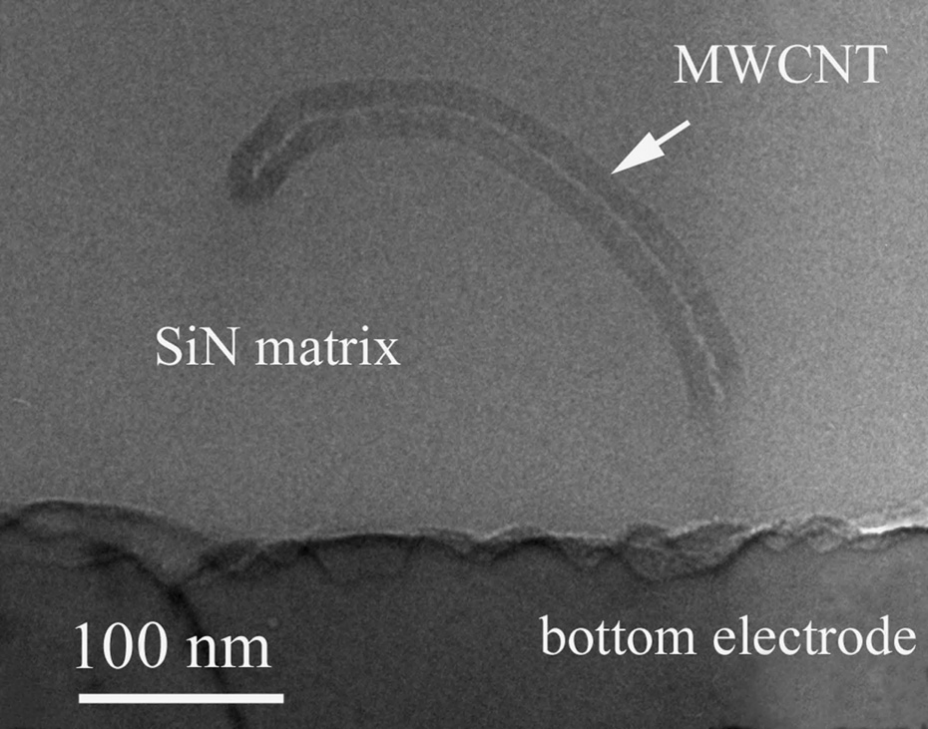
**XTEM image of a 30 nm diameter carbon nanotube embedded in the SiN matrix**. The image is taken in an area near the bottom electrode.

**Figure 5:**
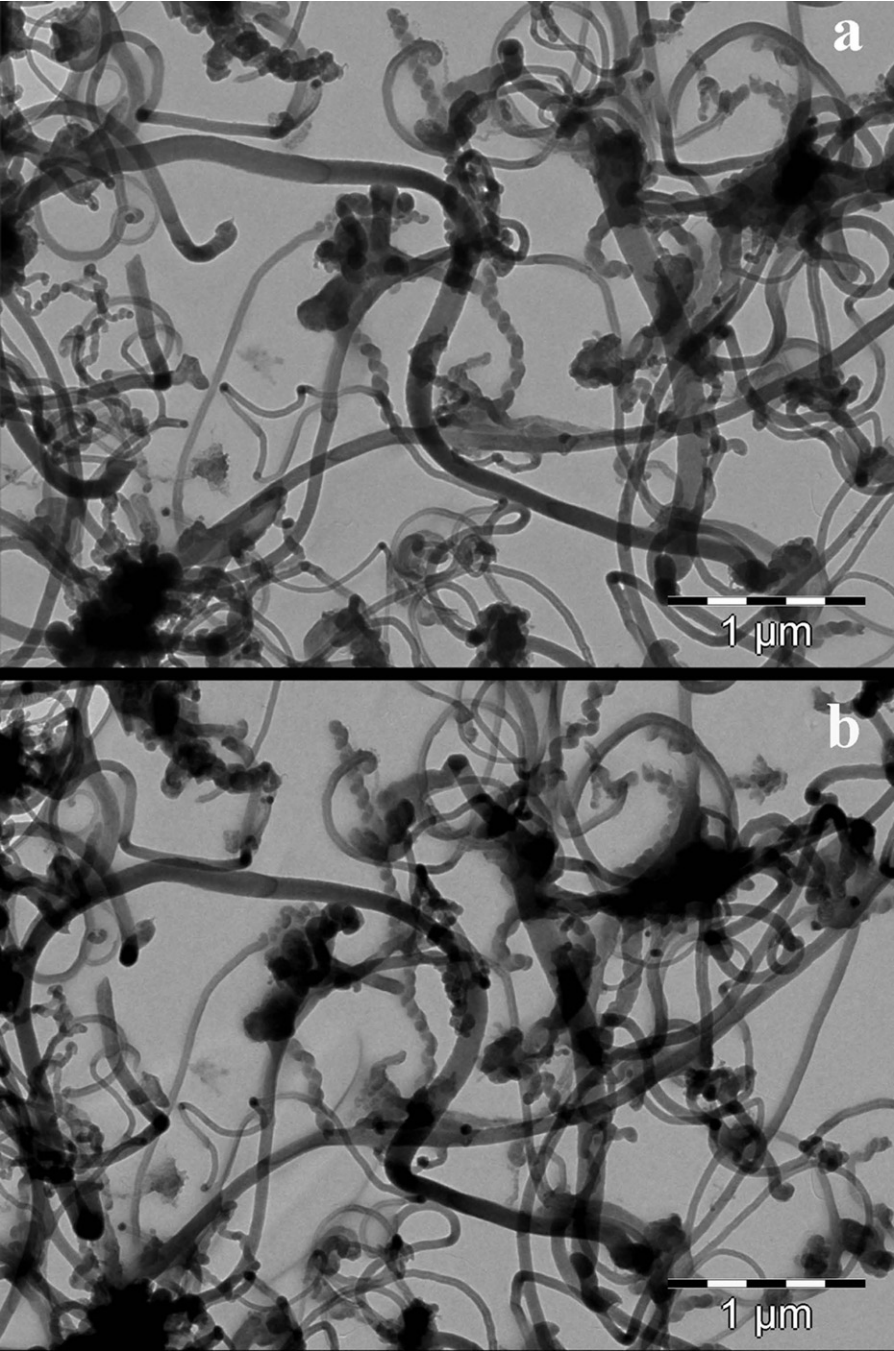
**TEM images of the MWCNT network deposited on the carbon TEM grid**. The image **(b) **is taken after the 30° tilting of the area shown in image **(a)**.

Looking at the XTEM specimen at higher magnification it was possible to observe the presence of the MWCNT in the SiN matrix. Figure 4 shows such a nanotube (about 30 nm thick) near the bottom electrode. We have to mention the difficulty to observe the MWCNTs embedded in amorphous SiN matrix by XTEM, for two reasons. First one, it is a low difference between the *Z *numbers of carbon, nitrogen, and silicon, which forms the structure. However, the 10-20 nm thick walls of the MWCNT show some low Bragg like contrast, coming from the graphitic like lattice planes, in the walls of the nanotube. This small contrast can be observed only in the very thin areas of the XTEM specimen, similar to the case presented in Figure 4. The second reason is the low density of the nanotubes network in the MWCNT-SiN layer. Additional information about the morphology of MWCNT network can be obtained if the nanotubes are pipetted directly onto a carbon-copper TEM grid, in a similar manner to that used for the sample preparation. Figure 5 shows a detail of such a spatial extension of MWCNT network formed on the carbon layer on the TEM grid. Using the high angle tilting of the microscope goniometer, we can show that such a network is uniformly extended in space (3D structure). Figure 5a,b shows the same area in the MWCNT network deposited on the carbon TEM grid. The image in Figure 5b is taken after the 30° tilting of the area shown in the Figure 5a. Analyzing the differences between these two images, we can estimate the depth of the network, which has the same order of magnitude as its lateral size.

We can suppose that such a CNT network keeps the same morphology during the deposition of the SiN matrix. The final XTEM specimen consists only in a slice of about 50 nm thick from the MWCNT network present in the SiN matrix. Consequently, in the XTEM specimen, the presence of MWCNTs will be rarely observed, in the very thin part of the specimen. However, the repetitive observations of the same XTEM specimen after a series of sequential small duration of ion milling allow us to observe different areas with MWCNT network embedded in the SiN matrix.

**Figure 6:**
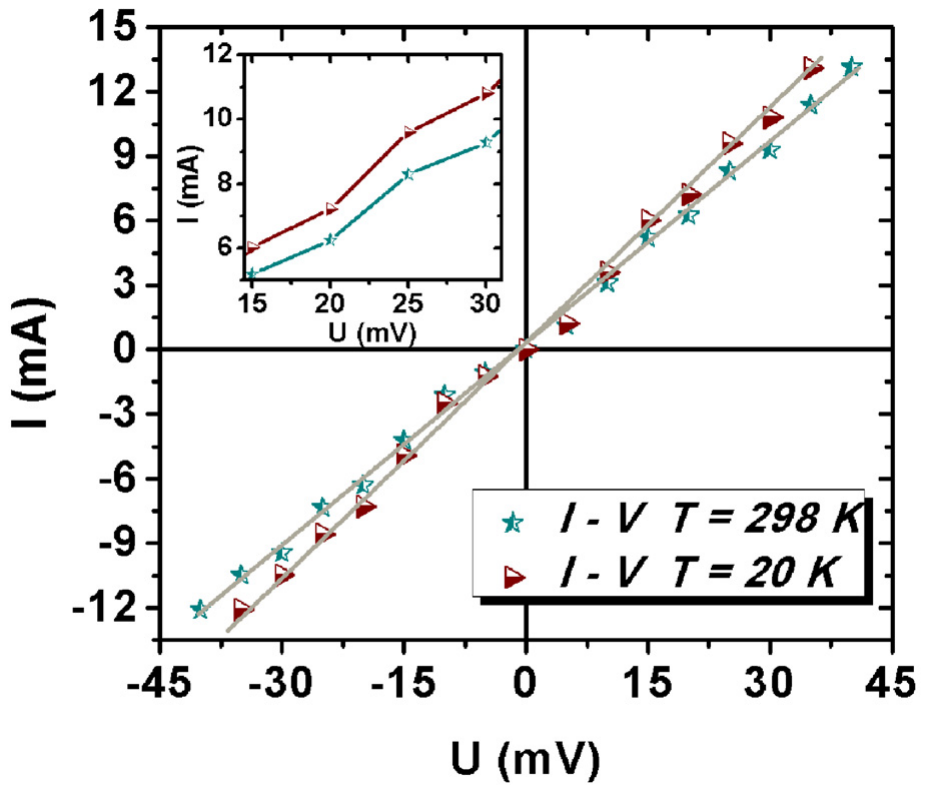
***I*-*V *characteristics taken at 298 and 20 K**. Inset: the region of the voltage percolation thresholds (*V *> 0).

Current-voltage characteristics are presented in Figure 6. They have practically a linear dependence, at both temperatures, typical for a metallic behavior. One can observe that the experimental points oscillate around the linear fit lines that give *G *≈ 0.31 S for *T *= 298 K and *G *≈ 0.36 S for *T *= 20 K.

**Figure 7:**
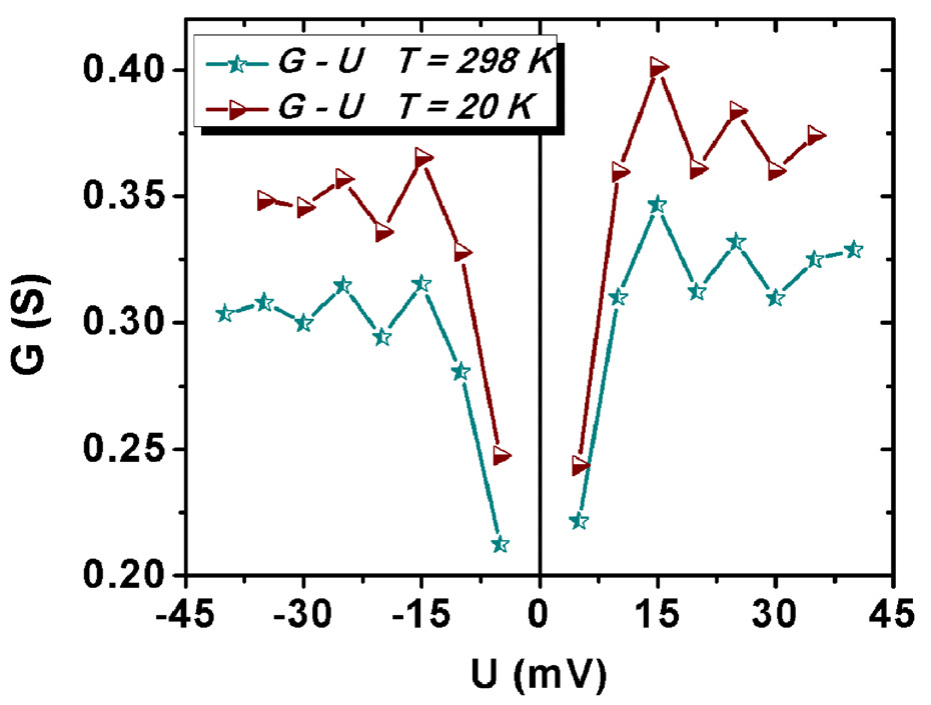
***G*-*V *characteristics taken at 298 and 20 K**.

To analyze these oscillations, the conductance-voltage curves were plotted (see Figure 7). These curves evidence that the maxima and minima of the conductance appear at the same voltages for both temperatures, namely, 15 and 25 mV for the maxima and 20 and 30 mV for the minima on both polarities. In our opinion, the conductance oscillations are due to percolation processes and the minima represent voltage percolation thresholds [25]. The percolation process in a disordered MWCNTs network is due to the field-assisted tunneling between neighboring nanotubes embedded in SiN. We assume that SiN fills up all the space in the structure. The interface between the nanotubes and the SiN matrix does not show any porosity (see Figure 4). The tunneling probability at the contact between MWCNTs varies as a function of their relative orientation and of the applied field. As the conduction through a metallic nanotube is quantified, it is expected that the current cannot increase continuously with the voltage. Therefore, the current-voltage curve tends to become sublinear [26] and the conductance reaches a minimum. When the electric field overpasses a critical value (that defines the voltage percolation threshold), the probability of the tunneling between convenient neighboring nanotubes increases enough to open less resistive paths. Then the current-voltage curve becomes superlinear and the conductance reaches a maximum. These minima and maxima are not periodically depending on the voltage and must be symmetric, meaning that they must appear at the same absolute value of the voltage for both bias polarities.

Conductance oscillations are previously presented in articles where they are attributed to Coulomb blockade effect [27,28], most of these results being observed in SWCNTs. The oscillations found by Ahlskog et al. [28] practically disappear when the sample temperature is increased from 4.6 to 20 K. On the other hand, the oscillations observed by LeRoy et al. [27] measured at 4.5 K are periodically depending on the voltage.

**Figure 8:**
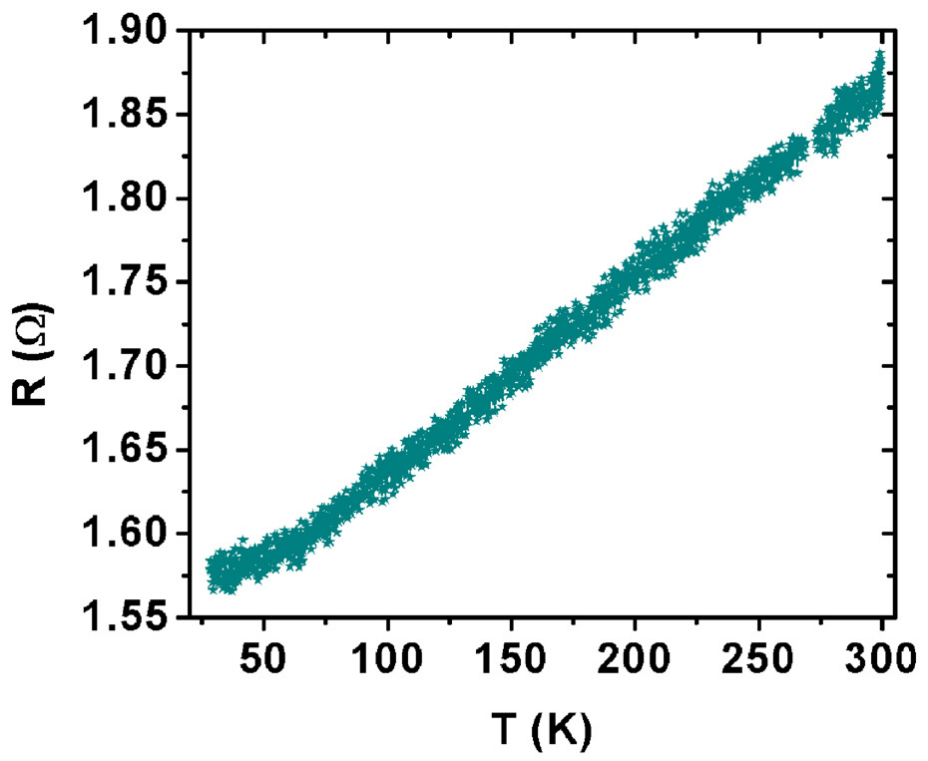
***R*-*T *characteristic taken at *U *= 20 mV**.

The oscillations observed in our measurements do not depend on the temperature and are not periodic. The resistance-temperature characteristic taken at *U *= 20 mV is presented in Figure 8. This characteristic is practically linear (except at low temperatures, under about 70 K). This is a supplementary argument for the metallic behavior of our MWCNTs network.

## Conclusions

The structure formed by the MWCNT network embedded in SiN was XTEM investigated. The TEM investigations, performed on nanotubes deposited directly on the carbon grid, reveal a uniform spatial extension of MWCNT network. In our opinion, this structure is preserved when MWCNT network is embedded in SiN.

The Cr/Al/MWCNT-SiN/Cr/Al samples present a metallic behavior, which is proved by the linear character of both the *I*-*V *and *R*-*T *characteristics.

The oscillations of the *I*-*V *and *G*-*V *curves are interpreted as due to percolation processes, as they are symmetric in bias polarization, are not periodic and are temperature independent. The voltage percolation thresholds of 20 and 30 mV on both bias polarities and both temperatures (20 and 298 K) are given by the conductance minima.

## Abbreviations

CNTs: carbon nanotubes; MWCNTs: multi-walled carbon nanotubes; PMMA: poly(methyl methacrylate); SWCNTs: single-walled carbon nanotubes; THF: tetrahydrofuran; XTEM: cross-sectional transmission electron microscopy.

## Competing interests

The authors declare that they have no competing interests.

## Authors' contributions

IS and AML carried out all electrical measurements and participated to modeling. VST carried out XTEM investigations. MLC conceived and coordinated the study, participated to modeling and drafted the manuscript. VI participated to modeling and writing the manuscript. MD carried out the design of the device. GK carried out the device fabrication. RB carried out the MWCNT deposition. All authors read and approved the final manuscript.
